# Hybrid Airway Management for Neuromonitoring-guided Selective Phrenic Nerve Surgery in Intractable Hiccups: A Case Report

**DOI:** 10.4274/TJAR.2026.262496

**Published:** 2026-10-30

**Authors:** Bekir Suat Kürkçüoğlu, Sinan Ünsal, Bahar Uslu Bayhan, Tuğçe Gazioğlu Kişi, Onur Bayrakçı

**Affiliations:** 1 University of Health Sciences Türkiye, Gaziantep City Hospital, Clinic of Anaesthesiology and Reanimation, Gaziantep, Türkiye; 2 University of Health Sciences Türkiye, Gaziantep City Hospital, Clinic of Thoracic Surgery, Gaziantep, Türkiye

**Keywords:** Intractable hiccups, phrenic nerve, intraoperative neuromonitoring, one-lung ventilation, electromyography endotracheal tube, bronchial blocker, sugammadex

## Abstract

Intractable hiccups are rare but debilitating, and may cause sleep disturbances, nutritional impairment, exhaustion, and a severe deterioration in quality of life. When pharmacological and interventional treatments fail, selected surgical procedures targeting the phrenic nerve may be considered. We report the anaesthetic management of a 32-year-old woman with idiopathic intractable hiccups who underwent right-sided selective phrenic nerve surgery guided by thoracoscopic neuromonitoring. Because thoracoscopic exposure and intraoperative neuromonitoring were required simultaneously, a hybrid airway strategy combining an electromyography endotracheal tube with a bronchial blocker was used. The patient was positioned in the left lateral decubitus position, and the bronchial blocker was placed in the right main bronchus under fiberoptic bronchoscopic guidance. One-lung ventilation was maintained for 35 minutes with FiO_2_ of 0.70 to 0.80, tidal volume of 5-6 mL kg^-1^, respiratory rate of 18 min^-1^, and PEEP of 5 cmH_2_O; the arterial blood gas analysis at 25 minutes showed pH 7.38, PaCO_2_ 41 mmHg, and PaO_2_ 92 mmHg. Sugammadex was administered intraoperatively to restore diaphragmatic motor responses required for branch-specific neuromonitoring. The medial and anterolateral branches of the right phrenic nerve exhibited abnormal electrophysiological responses and were treated with selective bipolar ablation and clipping. Postoperatively, the patient remained on room air, reported no intraoperative recall, and showed no clinically relevant diaphragmatic dysfunction on clinical and functional assessment. This case illustrates that established anaesthetic techniques can be carefully adapted to meet the competing requirements of lung isolation, anaesthetic depth control, and neuromonitoring in selected patients undergoing phrenic nerve surgery for intractable hiccups.

**Main Points**

• We report the anaesthetic management of a patient undergoing a phrenic nerve procedure for intractable hiccups using intraoperative electromyography (EMG) monitoring.

• An EMG endotracheal tube was utilized to ensure reliable neuromonitoring while maintaining secure airway control.

• A bronchial blocker was mandatorily employed to achieve lung isolation without compromising EMG signal integrity.

• This case demonstrates that a hybrid airway strategy combining an EMG tube and bronchial blocker may be feasible in complex procedures requiring simultaneous lung isolation and neuromonitoring.

## Introduction

Hiccups are involuntary, repetitive contractions of the diaphragm and intercostal muscles followed by abrupt glottic closure. Although most episodes are benign and self-limiting, persistent or intractable hiccups may cause sleep deprivation, nutritional impairment, exhaustion, psychological distress, and marked deterioration in quality of life. Intractable hiccups are generally defined as lasting longer than two months despite appropriate treatment.^1^

The hiccup reflex arc involves afferent pathways through the vagus, phrenic, and sympathetic nerves, central processing within the brainstem and cervical spinal cord, and efferent motor output mainly through the phrenic nerve to the diaphragm. Because this pathway is complex, etiological evaluation should include central nervous system, thoracic, and abdominal causes, as well as metabolic, pharmacological, and psychogenic causes. Pharmacological therapy is generally considered the first-line treatment and may include chlorpromazine, baclofen, gabapentin, metoclopramide, proton pump inhibitors, and other agents selected according to the suspected etiology.^2^ However, some patients remain refractory despite multimodal therapy.

Invasive interventions, including phrenic nerve block or surgical interruption, have been described in carefully selected refractory cases. However, proximal phrenic nerve interruption may cause ipsilateral diaphragmatic paralysis and reduced ventilatory reserve. In contrast, selective distal phrenic nerve procedures aim to target pathological branches while avoiding complete proximal phrenic nerve interruption.^3^ Such procedures require precise intraoperative localization of the relevant branch and, therefore, introduce specific anaesthetic challenges: maintaining lung isolation for thoracoscopic exposure, preserving or restoring neuromuscular transmission for phrenic nerve monitoring, avoiding intraoperative awareness during neuromuscular recovery, and maintaining oxygenation during one-lung ventilation.

This report describes the anaesthetic management of a patient undergoing right-sided thoracoscopic neuromonitoring-guided selective phrenic nerve surgery for idiopathic intractable hiccups. The educational focus is on adapting established anaesthetic tools—an electromyography (EMG) endotracheal tube, a bronchial blocker, total intravenous anaesthesia, bispectral index (BIS) monitoring, and intraoperative reversal of neuromuscular blockade—to meet the competing requirements of lung isolation and phrenic nerve neuromonitoring.

## Case Report

A 32-year-old woman presented with continuous hiccups lasting six months. The symptoms occurred at approximately 48 episodes per minute, causing severe sleep disturbance, nutritional impairment, exhaustion, and a marked deterioration in quality of life. A comprehensive etiological work-up was performed to exclude secondary causes of persistent hiccups. Neurological, thoracic, abdominal, gastrointestinal, metabolic, biochemical, and radiological evaluations did not reveal any identifiable organic cause. Therefore, the hiccups were considered idiopathic after exclusion of secondary etiologies.

The patient had previously received multiple pharmacological treatment protocols, including proton pump inhibitors, metoclopramide, antispasmodics, muscle relaxants, anxiolytics, chlorpromazine infusion, and baclofen. Despite these multimodal pharmacological treatments, the symptoms persisted. She also underwent consecutive right- and left-sided selective phrenic nerve blocks performed by the pain medicine unit for diagnostic and therapeutic purposes; however, these interventions did not provide sustained symptomatic improvement.

Dynamic fluoroscopic and ultrasonographic examinations demonstrated a pathological, asymmetric, paradoxical movement of the right hemidiaphragm, discordant with the left hemidiaphragm. Based on the failure of pharmacological and interventional treatments and the dynamic imaging findings, the right hemidiaphragm was considered the predominant pathological side, and right-sided thoracoscopic neuromonitoring-guided selective phrenic nerve surgery was planned.

The procedure was conducted in accordance with the principles of the Declaration of Helsinki. Written informed consent was obtained from the patient for the surgical procedure and for publication of this case report and accompanying images.

Preoperative anaesthetic evaluation revealed American Society of Anesthesiologists I status and Mallampati class I. Because intraoperative phrenic nerve neuromonitoring and thoracoscopic exposure were required simultaneously, a hybrid airway strategy was planned. A conventional double-lumen tube was avoided because it would not have provided the same compatibility with the planned neuromonitoring configuration. Therefore, an EMG endotracheal tube, combined with a bronchial blocker, was selected.

Standard monitoring, invasive arterial blood pressure monitoring, BIS monitoring, and neuromuscular monitoring were applied. Anaesthesia was induced with propofol 3 mg kg^-1^, fentanyl 1 μg kg^-1^, and rocuronium 0.6 mg kg^-1^. After adequate hypnotic depth was achieved, with a BIS value of approximately 45, the patient was intubated with a 7.5-mm EMG endotracheal tube. The patient was then positioned in the left lateral decubitus position with the right hemithorax up.

Anaesthesia was maintained using total intravenous techniques. Propofol was administered at 6-14 mg kg^-1^ h^-1^ and titrated to maintain BIS values between 40 and 60. Remifentanil was maintained at a constant rate of 0.2 μg kg^-1^ min^-1^ throughout the procedure. This anaesthetic strategy was selected to avoid interference with neuromonitoring and to maintain adequate hypnotic depth during periods of neuromuscular recovery.

Under fiberoptic bronchoscopic guidance, a 7-Fr bronchial blocker was advanced through the EMG endotracheal tube into the right main bronchus (Figure 1). The correct blocker position was confirmed bronchoscopically both after placement and after final positioning. One-lung ventilation was initiated with FiO_2_ 0.70-0.80, tidal volume of 5-6 mL kg^-1^ (approximately 350 mL), respiratory rate of 18 min^-1^, and PEEP of 5 cmH_2_O. Peripheral oxygen saturation remained between 95% and 100% during one-lung ventilation. One-lung ventilation lasted 35 minutes. An arterial blood gas analysis obtained at the 25^th^ minute of one-lung ventilation showed a pH of 7.38, a PaCO_2_ of 41 mmHg, and a PaO_2_ of 92 mmHg.

The most critical phase of anaesthetic management was the restoration of neuromuscular transmission before selective ablation and clipping, since diaphragmatic motor responses were required to identify the pathological distal branches of the phrenic nerve. Sugammadex 2 mg kg^-1^ was administered intraoperatively, 25 minutes after the induction dose of rocuronium. Although neuromuscular monitoring equipment was available, no documented train-of-four (TOF) or post-tetanic count (PTC) values prior to sugammadex administration could be retrieved on retrospective review. The purpose of reversal at this stage was not emergence or extubation but the restoration of sufficient diaphragmatic motor activity for intraoperative neuromonitoring.

During neuromuscular recovery after sugammadex administration, BIS values transiently increased to above 60 and were accompanied by mild increases in heart rate and arterial blood pressure. No gross patient movement was observed apart from the expected diaphragmatic contractions required for phrenic nerve monitoring. The propofol infusion was therefore increased by 2 mg kg^-1^ h^-1^, after which the BIS values were maintained within the target range of 40-60.

Intraoperative neuromonitoring was performed after careful dissection of the right phrenic nerve around the pericardium and exposure of its distal branching pattern near the diaphragmatic entry point. Four distal branches extending posteriorly, medially, anterolaterally, and laterally were identified. Spontaneous diaphragmatic electrical activity was recorded using conductive strip electrodes placed on the diaphragm. Additional conductive strip electrodes were then positioned along the intramuscular course of each distal phrenic nerve branch. Motor evoked potentials were elicited by primary motor cortex stimulation, and branch-specific responses were compared. The medial and anterolateral branches of the right phrenic nerve demonstrated markedly reduced amplitudes and delayed conduction compared with those of the posterior and lateral branches. These findings, together with the abnormal right hemidiaphragmatic movement observed during dynamic preoperative assessment, guided selective bipolar ablation and clipping of the medial and anterolateral branches.

After ablation and clipping, repeat assessment showed the disappearance of the pathological, hiccup-like diaphragmatic contractions. No additional neuromuscular blocking agent was administered after sugammadex because continued assessment of diaphragmatic motor responses was required during selective ablation, clipping, and the final confirmation phase of the procedure. The bronchial blocker was deflated, bilateral ventilation was restored, and recruitment maneuvers were performed. The total durations of surgery and anaesthesia were 115 and 155 minutes, respectively (Table 1).

At the end of the procedure, anaesthetic infusions were discontinued. Extubation was performed after the return of protective airway reflexes and the achievement of adequate clinical recovery. Complete resolution of hiccups was observed immediately after extubation. During postoperative evaluation, the patient was specifically questioned about intraoperative awareness and recall. She did not report recall, pain, choking sensation, distressing intraoperative experience, or awareness during surgery.

Postoperative recovery was uneventful. The patient was managed on room air and did not require supplemental oxygen or admission to intensive care. No clinical signs of respiratory compromise were observed. Early postoperative and pre-discharge chest radiographs did not show elevation of the right hemidiaphragm. Additional postoperative assessment included diaphragmatic ultrasonography, fluoroscopic sniff testing, pulmonary function testing, and clinical respiratory evaluation, none of which demonstrated clinically relevant diaphragmatic dysfunction. The patient was discharged on postoperative day seven. At the two-month follow-up, no recurrence of hiccups was reported.

## Discussion

This case illustrates the anaesthetic challenges encountered during right-sided thoracoscopic selective phrenic nerve surgery guided by neuromonitoring for idiopathic intractable hiccups. The main difficulty was not a single component of the anaesthetic technique, but rather the need to integrate several competing requirements: right lung isolation for thoracoscopic exposure, preservation of a neuromonitoring-compatible airway, restoration of diaphragmatic motor responses for phrenic nerve branch mapping, maintenance of adequate anaesthetic depth during neuromuscular recovery, and prevention of perioperative respiratory compromise. Therefore, the educational value of this case lies in the careful adaptation of established anaesthetic techniques to a rare surgical indication rather than in the introduction of an entirely new anaesthetic method.

Intractable hiccups may severely impair sleep, nutrition, physical endurance, and quality of life.^1, 2^ In the present case, the diagnosis was considered idiopathic only after a comprehensive etiological evaluation failed to identify an organic cause. Neurological, thoracic, abdominal, gastrointestinal, metabolic, biochemical, and radiological assessments did not reveal a secondary etiology. The patient also failed to respond to multimodal pharmacological therapy and bilateral diagnostic and therapeutic selective phrenic nerve blocks. Dynamic fluoroscopic and ultrasonographic examinations demonstrated a pathological, asymmetric, paradoxical movement of the right hemidiaphragm, discordant with the left hemidiaphragm. These findings support the selection of the right side for thoracoscopic selective phrenic nerve surgery.

The surgical approach was based on a recently described thoracoscopic neuroprotective phrenotomy technique for idiopathic incurable chronic hiccups.^3^ In this patient, intraoperative neuromonitoring was essential for identifying the pathological distal branches of the right phrenic nerve. After careful dissection around the pericardium, four distal branches of the right phrenic nerve were identified near the diaphragmatic entry point: posterior, medial, anterolateral, and lateral. Diaphragmatic electrical activity was recorded using conductive strip electrodes placed on the diaphragmatic muscle and along the intramuscular course of each distal branch. Motor evoked potentials were elicited by primary motor cortex stimulation, and branch-specific responses were compared. The medial and anterolateral branches demonstrated reduced amplitude and delayed conduction compared with the posterior and lateral branches. These findings, together with the abnormal right hemidiaphragmatic movement observed preoperatively, guided selective bipolar ablation and clipping of the medial and anterolateral branches. Repeat assessment after ablation and clipping showed the disappearance of the pathological hiccup-like diaphragmatic contractions.

Clarifying the neuromonitoring method is important because the role of the electromyographic endotracheal tube should not be misinterpreted. In this case, diaphragmatic electrical activity was recorded from conductive strip electrodes rather than from the endotracheal tube itself. The electromyographic endotracheal tube was used as part of a neuromonitoring-compatible airway strategy, thereby avoiding the need for a conventional double-lumen tube, which would have been less suitable for the planned monitoring configuration. This distinction is clinically relevant because the monitoring endpoint was not limited to visual assessment of diaphragmatic movement; it involved branch-specific electrophysiological comparison and confirmation of the functional effect of ablation.

The airway strategy also required careful planning. A conventional double-lumen tube would have provided reliable lung isolation but would not have allowed the same neuromonitoring-compatible configuration. Therefore, an electromyographic endotracheal tube was combined with a bronchial blocker. Similar combinations of an EMG endotracheal tube and bronchial blocker have been described for thoracic procedures requiring recurrent laryngeal nerve monitoring.^4^ In the present case, this established concept was adapted for right-sided thoracoscopic selective phrenic nerve surgery. The bronchial blocker was advanced into the right main bronchus under fiberoptic bronchoscopic guidance, enabling right lung isolation while maintaining the airway setup required for neuromonitoring.

The available oxygenation and ventilation data support the feasibility of this hybrid airway approach in this patient. One-lung ventilation lasted 35 minutes and was performed with FiO_2_ of 0.70-0.80, tidal volume of 5-6 mL kg^-1^, respiratory rate of 18 min^-1^, and PEEP of 5 cmH_2_O. Peripheral oxygen saturation remained between 95% and 100%. Arterial blood gas analysis obtained at the 25^th^ minute of one-lung ventilation showed pH 7.38, PaCO_2_ 41 mmHg, and PaO_2_ 92 mmHg. These findings indicate that adequate gas exchange was maintained during right lung isolation. However, additional respiratory mechanics data, such as peak airway pressure and plateau pressure, were not available for retrospective reporting.

Neuromuscular management was another critical aspect of this case. Rocuronium was required for induction, tracheal intubation, and initial airway manipulation. However, persistent neuromuscular blockade would have prevented reliable assessment of diaphragmatic motor responses during mapping of phrenic nerve branches. Previous physiological studies have shown that recovery from neuromuscular blockade may differ among the diaphragm, laryngeal muscles, and peripheral muscles.^5^ In this case, sugammadex 2 mg kg^-1^ was administered 25 minutes after the induction dose of rocuronium and before selective ablation and clipping. The purpose of reversal was not the emergence from anaesthesia, but the restoration of sufficient diaphragmatic motor activity for intraoperative neuromonitoring. No additional neuromuscular blocking agent was administered after administration of sugammadex, because continued assessment of diaphragmatic responses was required until the selective procedure was completed.

Nevertheless, this aspect of management has an important limitation. Sugammadex dosing should ideally be guided by quantitative neuromuscular monitoring. Although neuromuscular monitoring equipment was available, no documented TOF or PTC value before sugammadex administration could be retrieved retrospectively. This limitation has been explicitly acknowledged because the absence of an objective, documented neuromuscular measurement reduces reproducibility and limits generalizability. Future cases using this strategy should include quantitative neuromuscular monitoring and report TOF or PTC values before and after reversal.

The reversal phase required particular vigilance, because restoration of neuromuscular transmission during ongoing surgery may increase the risk of patient movement and intraoperative awareness if hypnotic depth is insufficient. Le Guen et al.^6^ reported that sugammadex administration during propofol and remifentanil anaesthesia may be associated with increases in BIS values suggestive of arousal. In the present case, BIS values transiently exceeded 60 after sugammadex administration and were accompanied by mild increases in heart rate and arterial blood pressure. No gross patient movement was observed apart from the expected diaphragmatic contractions required for neuromonitoring. The propofol infusion was increased by 2 mg kg^-1^ h^-1^, after which BIS values were maintained within the target range of 40-60. During postoperative evaluation, the patient was specifically questioned about intraoperative awareness and recall, and did not report pain, choking sensation, distressing intraoperative experiences, or any awareness or recall of events during surgery. However, a formal, structured awareness questionnaire was not used, which is a limitation.

The postoperative findings support the absence of clinically relevant diaphragmatic dysfunction after the selective procedure, although they should not be overinterpreted. The patient remained on room air and did not require supplemental oxygen or intensive care admission. No clinical signs of respiratory compromise were observed. Early postoperative and pre-discharge chest radiographs showed no elevation of the right hemidiaphragm. Additional postoperative evaluation, including diaphragmatic ultrasonography, fluoroscopic sniff testing, pulmonary function testing, and clinical respiratory assessment, did not demonstrate clinically relevant diaphragmatic dysfunction. This postoperative course contrasts with the potential risk of diaphragmatic paralysis after more proximal interruption of the phrenic nerve. A previous case report described phrenic nerve clipping for intractable hiccups after stroke,^7^ but the present case differs by emphasizing branch-specific neuromonitoring, selective distal treatment, and the anaesthetic strategy required to support this approach.

This report has several limitations. First, it represents a single case and cannot establish the safety or efficacy of this strategy. Second, no documented TOF or PTC values were available before sugammadex administration. Third, although the patient was specifically questioned postoperatively and denied awareness or recall, a formal structured awareness questionnaire was not used. Fourth, although postoperative clinical, radiographic, ultrasonographic, fluoroscopic, and pulmonary function assessments did not demonstrate clinically relevant diaphragmatic dysfunction, the detailed quantitative diaphragmatic excursion and pulmonary function values were not presented. Finally, some respiratory mechanics data during one-lung ventilation, including peak and plateau airway pressures, were not available. Despite these limitations, this case provides a practical example of how established anaesthetic techniques may be integrated for a rare procedure requiring simultaneous lung isolation, anaesthetic depth control, neuromuscular recovery, and phrenic nerve neuromonitoring.

## Conclusion

Right-sided thoracoscopic selective phrenic nerve surgery for intractable hiccups requires careful coordination of lung isolation, anaesthetic depth, neuromuscular management, and intraoperative neuromonitoring. In this case, a hybrid airway strategy using an EMG endotracheal tube and a bronchial blocker, combined with total intravenous anaesthesia, BIS monitoring, and procedural sugammadex-mediated recovery of diaphragmatic motor responses, enabled neuromonitoring-guided selective ablation and clipping of pathological distal phrenic nerve branches. The patient had immediate resolution of hiccups and no clinically relevant postoperative diaphragmatic dysfunction. This single case suggests that established anaesthetic techniques can be adapted for selected patients undergoing this rare procedure; however, future reports should include quantitative neuromuscular monitoring, structured awareness assessment, and detailed postoperative diaphragmatic function measurements.

## Ethics

**Informed Consent:** Written informed consent was obtained from the patient for publication of this case report and accompanying images. The patient was informed that personal identifying information would not be disclosed.

## Figures and Tables

**Figure 1:**
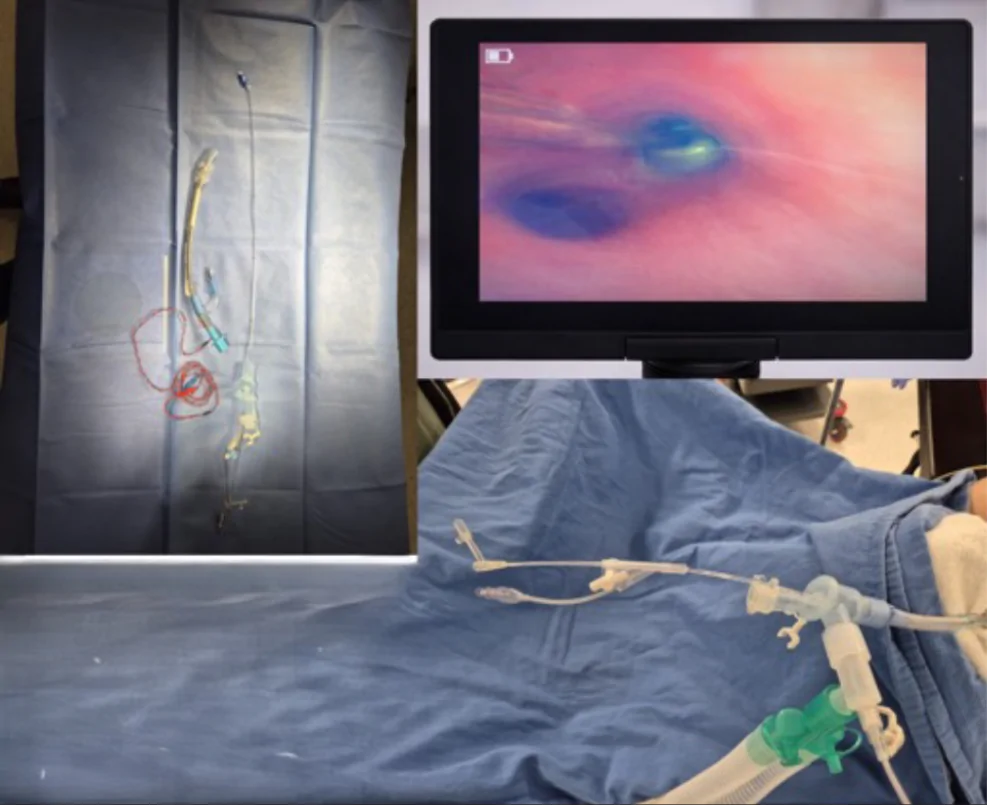
Hybrid airway setup for right-sided thoracoscopic neuromonitoring-guided selective phrenic nerve surgery. The image demonstrates the configuration of an electromyographic endotracheal tube and a 7-Fr bronchial blocker, together with confirmation of bronchial blocker placement in the right main bronchus by fiberoptic bronchoscopy. This setup enabled right lung isolation for thoracoscopic exposure while maintaining compatibility with the intraoperative neuromonitoring strategy.

**Table 1. Summary of Perioperative Management and Key Outcomes:** 

**Domain**	**Key details**
Preoperative status	Six-month idiopathic intractable hiccups refractory to multimodal pharmacological treatment and bilateral selective phrenic nerve blocks; no organic cause identified after comprehensive evaluation.
Side selection	Dynamic fluoroscopic and ultrasonographic assessment showed pathological asymmetric paradoxical movement of the right hemidiaphragm.
Airway and positioning	Left lateral decubitus position with the right hemithorax up; 7.5-mm electromyography endotracheal tube and 7-Fr bronchial blocker placed in the right main bronchus under fiberoptic bronchoscopy.
One-lung ventilation	Duration 35 minutes; FiO_2_ 0.70 to 0.80, tidal volume 5-6 mL kg^-1^, respiratory rate 18 min-1, PEEP 5 cmH_2_O, SpO_2_95-100%; ABG at 25 minutes: pH 7.38, PaCO_2_ 41 mmHg, PaO_2_ 92 mmHg.
Neuromonitoring and reversal	Sugammadex 2 mg kg-1 was administered 25 minutes after rocuronium to restore diaphragmatic motor responses; medial and anterolateral branches showed reduced amplitude and delayed conduction.
Surgical intervention	Neuromonitoring-guided selective bipolar ablation and clipping of the medial and anterolateral branches of the right phrenic nerve; pathological hiccup-like contractions disappeared after treatment.
Outcome	Surgery 115 minutes, anaesthesia 155 minutes; no supplemental oxygen or ICU admission; no intraoperative recall; no clinically relevant postoperative diaphragmatic dysfunction; no recurrence at two months.

ICU, intensive care unit; ABG, arterial blood gas
